# Supplementary material for: Interpreting mosquito feeding patterns in Australia through an ecological lens: an analysis of blood meal studies
Source: Parasit Vectors. 2019 Apr 4;12:156. doi: 10.1186/s13071-019-3405-z (PMC6448275; doi:10.1186/s13071-019-3405-z)
Supplement: Supplementary file 1 — Additional file 1: Table S1. Reported blood-meal results for Australian mosquito species. Numbers in the columns representing number of blood meals for each vertebrate. Table S2. Derivation of 2 × 2 contingency tables for mosquito feeding preferences. [file 13071_2019_3405_MOESM1_ESM.docx]

| **Additional file 1: Table S1.** **Reported blood meal results for Australian mosquito species. Numbers in the columns representing number of blood meals for each vertebrate.** | | | | | | | | | | | | | | | | | |
| --- | --- | --- | --- | --- | --- | --- | --- | --- | --- | --- | --- | --- | --- | --- | --- | --- | --- |
| **Mosquito species** | **Reference studies** | **Human** | **Dog** | **Cat** | **Horse** | **Bird** | **Possum** | **Marsupial** | **Flying fox** | **Cattle** | **Pig** | **Rabbit** | **Sheep** | **Goat** | **Fox** | **Rat** | **Total** |
| Ae aegypti | [22] | 131 | 23 | 2 | 0 | 10 | - | 0 | - | 0 | - | 1 | - | - | - | 0 | **174** |
| Ae alboannualtus | [31] | 2 | - | - | - | - | - | 2 | - | - | - | 1 | - | - | - | - | **5** |
| Ae alternans | [31] | - | - | - | - | - | - | - | - | 1 | - | - | - | - | - | - | **1** |
| Ae camptorhynchus | [23] | 5 | 0 | 0 | - | 7 | 2 | - | - | 83 | - | - | 21 | 2 | 2 | 1 | **125** |
| Ae lineatopennis | [22]  [29] | 0 | 1 | 0 | 0 | 0 | - | 3 | - | 5 | - | 0 | - | - | - | 0 | **9** |
| Ae multiplex | [22] | 0 | 0 | 0 | 0 | 0 | - | 1 | - | 0 | - | 0 | - | - | - | 0 | **1** |
| Ae normanensis | [30]  [29]  [33] | 1 | 3 | 0 | 17 | 5 | - | 54 | - | 21 | 2 | - | - | - | - | - | **104** |
| Ae notoscriptus | [23]  [22]  [30]  [21] | 38 | 63 | 4 | 0 | 32 | 44 | 16 | 4 | 2 | 0 | 0 | 0 | 0 | 0 | 2 | **207** |
| Ae procax | [22]  [21] | 3 | 17 | 0 | 1 | 8 | 0 | 5 | 0 | 2 | - | 0 | - | - | - | 0 | **36** |
| Ae queenslandis | [31] | 5 | - | - | - | - | - | 24 | - | - | - | - | - | - | - | - | **29** |
| Ae reesi | [29] | 0 | 0 | - | 0 | - | - | 1 | - | 1 | - | - | - | - | - | - | **2** |
| Ae subauridorsum | [31] | 1 | - | - | - | - | - | - | - | - | - | - | - | - | - | - | **1** |
| Ae theobaldi | [31] | 1 | - | - | - | 1 | - | - | - | - | - | 2 | - | - | - | - | **4** |
| Ae vigilax | [32]  [22]  [30]  [21]  [31]  [29]  [33] | 37 | 47 | 3 | 25 | 45 | 12 | 30 | 1 | 37 | 2 | 30 | - | - | - | 6 | **278** |
| Ae vittiger | [21]  [31]  [33] | 6 | 2 | 0 | 3 | 1 | 1 | 1 | 0 | 1 | 0 | - | - | - | - | - | **15** |
| An amictus | [30]  [29] | 0 | 3 | 0 | 0 | 0 | - | 0 | - | 2 | 0 | - | - | - | - | - | **5** |
| An annulipes | [23]  [22]  [30]  [31]  [29] | 41 | 187 | 18 | 29 | 62 | 0 | 104 | - | 276 | 3 | 579 | 0 | 0 | 0 | 0 | **1332** |
| An bancroftii | [30]  [29] | 28 | 26 | 3 | 2 | 2 | - | 12 | - | 18 | 1 | - | - | - | - | - | **93** |
| An farauti | [30]  [29] | 0 | 1 | 0 | 0 | 0 | - | 2 | - | 6 | 0 | - | - | - | - | - | **9** |
| An hilli | [30] | 1 | 2 | 0 | 0 | 0 | - | 0 | - | 0 | 0 | - | - | - | - | - | **3** |
| An meraukensis | [29] | 0 | 0 | - | 0 | - | - | 0 | - | 2 | - | - | - | - | - | - | **2** |
| An novaguinensis | [29] | 0 | 0 | - | 0 | - | - | 0 | - | 6 | - | - | - | - | - | - | **6** |
| An stigmaticus | [31] | - | - | - | - | - | - | 2 | - | - | - | - | - | - | - | - | **2** |
| Cq linealis | [23]  [22]  [21] | 4 | 3 | 0 | 4 | 7 | 1 | 8 | 0 | 1 | - | 10 | 0 | 0 | 0 | 2 | **41** |
| Cq xanthogaster | [22]  [21]  [29] | 0 | 15 | 1 | 5 | 17 | 0 | 10 | 0 | 7 | - | 0 | - | - | - | 0 | **55** |
| Cx annulirostris | [23]  [32]  [27]  [22]  [24]  [30]  [21]  [31]  [29]  [33] | 183 | 673 | 42 | 258 | 503 | 57 | 2884 | 1 | 808 | 347 | 143 | 11 | 0 | 0 | 44 | **6089** |
| Cx australicus | [22]  [21] | 0 | 1 | 0 | 5 | 17 | 2 | 0 | 0 | 0 | - | 0 | - | - | - | 0 | **25** |
| Cx bitaeniorhynchus | [30]  [29]  [33] | 0 | 3 | 0 | 0 | 2 | - | 6 | - | 0 | 0 | - | - | - | - | - | **11** |
| Cx fatigans | [31] | 54 | 12 | - | 15 | 197 | - | - | - | 2 | - | - | - | - | - | - | **280** |
| Cx globocoxitus | [23] | 2 | 0 | 2 | - | 9 | 0 | - | - | 1 | - | - | 0 | 0 | 0 | 0 | **14** |
| Cx halifaxii | [22] | 0 | 0 | 0 | 0 | 1 | - | 0 | - | 0 | - | 0 | - | - | - | 0 | **1** |
| Cx hilli | [22] | 3 | 1 | 0 | 0 | 0 | - | 0 | - | 0 | - | 0 | - | - | - | 0 | **4** |
| Cx Lophoceramyia sp | [30]  [29] | 10 | 6 | 3 | 0 | 5 | - | 0 | - | 3 | 0 | - | - | - | - | - | **27** |
| Cx molestus | [23]  [22] | 3 | 1 | 0 | 0 | 10 | 0 | 0 | - | 3 | - | 0 | 0 | 0 | 0 | 0 | **17** |
| Cx orbostiensis | [22] | 1 | 0 | 0 | 0 | 1 | - | 0 | - | 1 | - | 0 | - | - | - | 0 | **4** |
| Cx palpalia | [33] | 0 | 2 | 0 | 0 | 1 | - | 12 | - | 1 | 0 | - | - | - | - | - | **16** |
| Cx pullus | [22]  [29] | 1 | 0 | 0 | 0 | 1 | - | 2 | - | 2 | - | 0 | - | - | - | 0 | **6** |
| Cx quinquefasciatus | [23]  [22]  [30]  [21]  [31]  [29]  [33] | 398 | 2417 | 73 | 93 | 1514 | 2 | 35 | 0 | 49 | 58 | 24 | 0 | - | 0 | 0 | **4746** |
| Cx sitiens | [32]  [22]  [21]  [33] | 6 | 8 | 0 | 3 | 39 | 0 | 13 | 0 | 1 | 2 | 2 | - | - | - | 1 | **77** |
| Cx squamosus | [30] | 0 | 13 | 2 | 2 | 62 | - | 2 | - | 1 | 0 | - | - | - | - | - | **84** |
| Cx starckeae | [30] | 0 | 3 | 1 | 0 | 4 | - | 2 | - | 1 | 0 | - | - | - | - | - | **11** |
| Cx whitmorei | [33] | 0 | 0 | 0 | 0 | 0 | - | 1 | - | 0 | 1 | - | - | - | - | - | **2** |
| Ma linealis | [31] | - | - | - | - | 1 | - | - | - | - | - | - | - | - | - | - | **1** |
| Ma septempunctata | [33] | 0 | 0 | 0 | 0 | 1 | - | 3 | - | 0 | 0 | - | - | - | - | - | **4** |
| Ma uniformis | [22]  [30]  [29]  [33] | 25 | 4 | 1 | 0 | 2 | - | 5 | - | 22 | 1 | 0 | - | - | - | 0 | **60** |
| U albescens | [30] | 0 | 0 | 0 | 0 | 0 | - | 1 | - | 0 | 0 | - | - | - | - | - | **1** |
| Ve carmenti | [22] | 1 | 1 | 0 | 0 | 0 | - | 4 | - | 0 | - | 0 | - | - | - | 0 | **6** |
| Ve funerea | [22]  [33] | 3 | 0 | 0 | 0 | 0 | - | 0 | - | 0 | 0 | 0 | - | - | - | 0 | **3** |
| **Grand Total** |  | **994** | **3538** | **155** | **462** | **2567** | **121** | **3245** | **22** | **1366** | **417** | **792** | **32** | **2** | **2** | **56** | **14044** |

| **Additional file 1: Table S2.** Derivation of 2x2 contingency tables for mosquito feeding preferences | | | |
| --- | --- | --- | --- |
|  | Focal host taxon | All other hosts | Totals |
| Focal mosquito species | ***a*** | *A-a* | ***A*** |
| All other mosquito species | *B-a* | *N-A-B+a* | *N-A* |
| Totals | ***B*** | *N-B* | ***N*** |

***a*** Number of records of the focal mosquito species feeding on the focal host taxon

***A*** Total number of records for the focal mosquito species

***B*** Total number of records for the focal host taxon

***N*** Overall number of records

Values in grey shaded cells are obtained by subtraction
